# LRG1 May Accelerate the Progression of ccRCC via the TGF-*β* Pathway

**DOI:** 10.1155/2020/1285068

**Published:** 2020-03-28

**Authors:** Quan Hong, Shuqiang Wang, Shuxin Liu, Xiangmei Chen, Guangyan Cai

**Affiliations:** ^1^Medical School of Chinese PLA, Beijing 100853, China; ^2^Department of Nephrology, Chinese PLA General Hospital, Chinese PLA Institute of Nephrology, State Key Laboratory of Kidney Diseases, National Clinical Research Center for Kidney Diseases, Beijing Key Laboratory of Kidney Diseases, Beijing 100853, China; ^3^Dalian Municipal Central Hospital, Dalian 116033, China

## Abstract

Clear cell renal cell carcinoma (ccRCC) accounts for 60-70% of renal cell carcinoma (RCC) cases. It is an urgent mission to find more therapeutic targets for advanced ccRCC. Leucine-rich a-2-glycoprotein 1 (LRG1) is a secreted protein associated with a variety of malignancies. Our study focused on the expression and mechanism of LRG1 in ccRCC based on data from The Cancer Genome Atlas (TCGA) and provided primary verification including LRG1 expression detection, LRG1 gene methylation detection, and downstream signaling detection. We found that LRG1 was overexpressed in ccRCC kidney tissue samples, and the methylation level of LRG1 gene was significantly decreased in ccRCC. Moreover, the expression of LRG1 was negatively related to patient survival. Based on our previous study and the verification reported in this article, we propose that demethylation-induced overexpression of LRG1 is likely to accelerate ccRCC progression via the TGF-*β* pathway.

## 1. Introduction

Leucine-rich a-2-glycoprotein 1 (LRG1) is a secreted glycoprotein with a molecular weight of ~50 kDa that belongs to the leucine-rich repeat protein family. The expression of LRG1 is associated with a variety of malignancies, such as non-small-cell lung cancer (NSCLC) [1], ovarian cancer [2], and bladder cancer [3], and is thought to promote tumor growth via the angiogenesis process. Clear cell renal cell carcinoma (ccRCC) is the most common renal cell carcinoma (RCC) and accounts for 60-70% of RCC cases. Early-stage ccRCC can be clinically cured by surgical excision, but many patients are first diagnosed with advanced ccRCC and have to accept chemotherapy. Most of the drugs currently on the market target VEGF and VEGFR but have limited effects on advanced ccRCC [4].

LRG1 has been indicated to promote neovascularization in mouse models of ocular disease by potentiating endothelial TGF-*β*/activin receptor-like kinase 1 (ALK1) signaling [5]. Our previous study showed that LRG1 promoted diabetic kidney disease (DKD) progression by enhancing TGF-*β*-induced angiogenesis [6]. In this study, we found that LRG1 was closely related to ccRCC. LRG1 accelerates the progression of CCRCC via the TGF-*β* pathway.

## 2. Methods and Materials

### 2.1. Clinical Cohorts and RNA-Seq Data

RNA-seq data and clinical cohorts were obtained from The Cancer Genome Atlas (TCGA; http://www.tcga.org/). A total of 528 ccRCC patients and 72 normal controls were included in the analysis. The clinical data included patient age, sex, race, and survival time.

### 2.2. Analysis of RNA-Seq Data

Differential expression analysis comparing the normal controls and ccRCC patients and Kaplan-Meier survival curve analysis were conducted with the Human Protein Atlas (https://www.proteinatlas.org), UALCAN analysis tools (http://ualcan.path.uab.edu/) [7], and SPSS 22.0. Bioinformatic analysis of correlated genes included gene ontology (GO) and protein-protein interaction (PPI) analyses with Metascape analysis tools (http://metascape.org/) [8] and the Cbioportal for cancer genomics (http://www.cbioportal.org/) [9]. Methylation analysis was conducted with MethHC (http://methhc.mbc.nctu.edu.tw) [10]. All these analysis tools are publicly available online.

### 2.3. Sample Collection

Our study was approved by the institutional research ethics committee of the Chinese People's Liberation Army (PLA) General Hospital (No. S2015-061-01). All procedures performed in this study involving human participants were conducted in accordance with the ethical standards of the institutional and national research committees and either the 1964 Declaration of Helsinki and its later amendments or comparable ethical standards. All of the samples were obtained from the Nephrology Department of the Chinese PLA General Hospital. A total of 6 paired cancer and adjacent tissue samples from RCC patients harvested via nephrectomy were included in the study as a discovery cohort.

### 2.4. qPCR Detection

The First Strand cDNA Synthesis Kit (New England Biolabs, E6560S) was used for cDNA synthesis with a standard protocol. Each RNA/d(T)23VN sample was predenatured for 5 minutes at 65°C, the 20-*μ*l cDNA synthesis reaction was incubated at 42°C for one hour, and the enzyme was inactivated at 80°C for 5 minutes.

The SYBR Select Master Mix Kit (Applied Biosystems, 4472908) was used for PCR, which was performed with a standard procedure on an Applied Biosystems 7500 Real-Time PCR system.

Primer used in this study: LRG-1: forward, 5′-GGACACCCTGGTATTGA AAGAAA-3′; reverse, 5′-TAGCCGTTCTAATTGCAGCGG-3′. 18S: 5′-GTAACC CGTTGAACCCCATT-3′; reverse, 5′-CCATCCAACGGTAGTAGCG-3′.

### 2.5. Western Blot Detection

For LRG1 detection, 50-mg kidney tissue samples were harvested with a lysis buffer (Beyotime, P0013B). Protein concentrations were determined with the BCA assay (Thermo Pierce, 23225), and proteins were boiled in an LDS sample buffer (Invitrogen, NP0007) for 10 minutes. A total of 30 *μ*g protein were resolved by 10% SDS-polyacrylamide gel electrophoresis and then transferred to nitrocellulose (NC) membranes (Pall BioTrace, 66485). The membranes were blocked for 1 hour with 5% fat-free skim milk in Tris-buffered saline and incubated with a primary antibody diluted 1 : 1,000 overnight at 4°C. The membranes were rinsed with Tris-buffered saline containing 0.1% Tween 20 three times for 7 minutes each time the next day and incubated with a secondary antibody diluted 1 : 2,000 for 2 hours. Finally, the proteins on the membranes were detected with a chemiluminescent reagent after rinsing again.

The following antibodies were used for Western blotting: primary antibodies against LRG1 (ABCAM, ab178698, Rab), TGF-*β* (Abcam, ab92486, Rab), and GAPDH (CST, 2118, Rab) and a goat anti-rab IgG-HRP secondary antibody (Beyotime, A0208). Proteins were detected with BeyoECL Plus (Beyotime, P0018s) chemiluminescent reagent.

### 2.6. Cell Culture

The expression of LRG1 and TGF-*β* in the 786-O cancer cell line (ATCC, CRL-1932) was verified. Cells were cultured in RPMI-1640 medium (ATCC, 30-2001) supplemented with 10% fetal bovine serum (FBS; Corning, 35-010-CV) and 1% penicillin-streptomycin (Corning, 30-002-CI) at 37°C in a humidified 5% CO2 environment.

### 2.7. Cytokine Intervention

Cells were seeded in 6-well plates and synchronized for 12 hours at 60% confluence. Then, the culture medium was replaced with RPMI-1640 supplemented with 2% FBS and LRG1 (10 ng/ml) or heat-denatured LRG1 (10 ng/ml) to exclude the endotoxin influence, and the cells were harvested with TRIzol after 24 hours. The cytokines used in the study were carrier-free recombinant human LRG1 (R&D, 7890-LR).

### 2.8. LRG1 Knockdown

789-O cells were reseeded in 6-well plates before siRNA transfection. siRNA (2.5 *μ*g) transfection was performed using Lipofectamine 2000 reagent (10 *μ*l per well). The medium was changed to fresh complete medium after 6 hours, and the cells were harvested with TRIzol reagent (Thermo Fisher Scientific, 15596026) after 24 hours. The sequences of the siRNA oligos for LRG1 were sense, 5′-CCUCUAAGCUCCAAGAAUUTT-3′ and antisense, 5′-AAUUCUUGGAGCUUAGAGGTT-3′ [11].

### 2.9. Pyrosequencing

Pyrosequencing was used to determine DNA methylation level at individual cytosines (CpGs) in DNA extracted from paracarcinoma and ccRCC carcinoma samples. EZ DNA Methylation Kit (Zymo Research, D5001) were used to perform bisulfite conversion with five hundred nanograms of DNA. Converted DNA was amplified using the AmpliTaq Gold 360 buffer Kit (Applied Biosystems, 4398853) and then the promoter methylation of LRG1 gene was detected by PCR with primers mapping to the homologous promoter CpG1 and CpG2 regions of LRG1. PCR primers are shown in Table 1. Pyrosequencing was performed using PyroMark Q24Gold reagents (Qiagen, 970802), and data were analyzed by PYROMARK Q24 1.0.10 software (Qiagen). Background nonconversion levels were <3%.

### 2.10. Statistical Analysis of Verification

Experimental data are presented as the mean ± SD. Data were analyzed with SPSS 22.0 software. A two-tailed Student's *t*-test was used to analyze differences in experimental data between two groups. *p* < 0.05 was considered statistically significant.

## 3. Results

### 3.1. Clinical ccRCC Patient Data

We downloaded clinical data for ccRCC patients (Table 2) from the TCGA and checked the data by using the Human Protein Atlas and UALCAN analysis tools. A total of 528 ccRCC patients, including 344 males and 184 females with an average age of 60.54 ± 12.14 years old, were included in the study. The ratio of males to females was approximately 2 : 1. A total of 49.8% (263/528) of the patients were first diagnosed at stage 1, and 355 patients are still alive while 173 patients had died, with median survival times of 1,540.7 days and 965.8 days, respectively, at the time of data collection. This is close to the natural population morbidity of ccRCC; thus, the data are able to represent the essential features of ccRCC.

### 3.2. LRG1 Is Overexpressed in CCRCC

**Figure 1:**
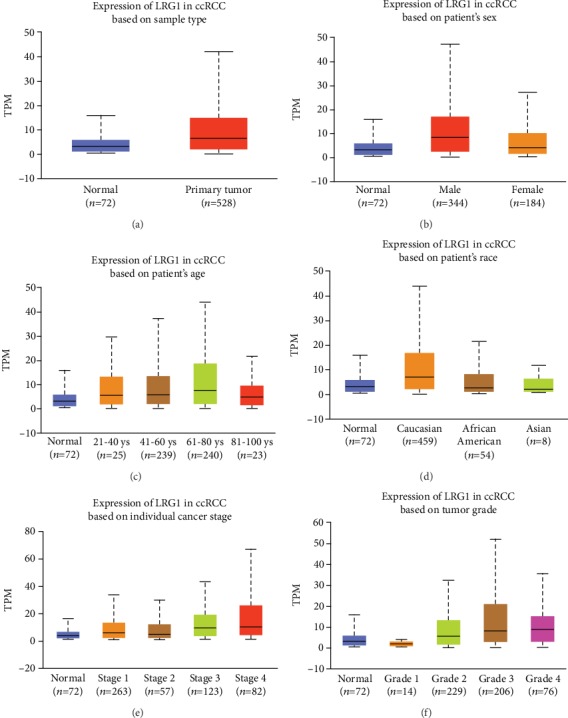
The differential expression of LRG1 in ccRCC patients. (a) LRG1 is upregulated in primary tumor (*p* < 0.001). (b) LRG1 expression of male patients is significantly different from male and normal contols (*p* < 0.001). (c) LRG1 expression was significantly different between the normal control group and the 41-60 (*p* = 0.0024) and 61-80-year-old subgroups (*p* < 0.0001). (d) Caucasian group is the most differential expressed group compared with normal controls (*p* < 0.0001). (e) LRG1 expression levels were significantly different between the normal control group and different ccRCC stage subgroups (*p* < 0.0001). (f) All grades except grade 1 were significantly different from that in normal controls (*p* < 0.05). ^∗^ccRCC: clear cell renal cell carcinoma; TPM: transcript per million.

LRG1 was significantly overexpressed in ccRCC patients compared with normal controls (Figure 1(a)). The transcript per million (TPM) levels in the normal controls and ccRCC patients were 3.187 (1.294, 5.612) and 6.504 (2.099, 14.882), respectively (*p* < 0.001). [median (lower quartile, upper quartile)]. To analyze the expression of LRG1 in detail, we classified LRG1 expression in ccRCC patients stratified by patient sex, age, race, grades, or cancer stage.

#### 3.2.1. Sex

The LRG1 expression in male patients was significantly different from that in normal controls and female patients (*p* < 0.001) (Figure 1(b)). In contrast, the female patients were not significantly different from the normal controls (*p* = 0.097) (Figure 1(b)). According to these data, we hypothesized that LRG1 is more important in male patients than in female patients.

#### 3.2.2. Age

Patients were divided into 4 subgroups by age with a unit of 20 years including subgroups of 21-40 years old, 41-60 years old, 61-80 years old, and 81-100 years old. Patients in the 41-80-year-old subgroups accounted for 90.72% of the total (Table 2). Among all 4 groups, LRG1 expression levels were significantly different between the normal control group and the 41-60 (*p* = 0.0024) and 61-80-year-old subgroups (*p* < 0.0001) (Figure 1(c)), which is in accordance with the highest risk age for ccRCC.

#### 3.2.3. Race

LRG1 expression in normal controls was significantly different from that in Caucasian ccRCC patients (*p* < 0.0001) but not from that in African-American or Asian patients (Figure 1(d)). Moreover, the expression in the Caucasian patients was significantly different from that in the African-born American patients (*p* = 0.0089) and Asian patients (*p* = 0.011) (Figure 1(d)). This indicated to us that LRG1 might be a more meaningful marker in Caucasian ccRCC patients than in patients of other ethnicities.

#### 3.2.4. Cancer Stage

LRG1 expression levels were significantly different between the normal control group and different ccRCC stage subgroups, such as the normal control group compared with the stage I, stage III, or stage IV subgroup (Figure 1(e)), and these three groups contained 88.6% of all patients.

#### 3.2.5. Tumor Grade

Patients were classified into four grades at first diagnosis. LRG1 expression in all grades except grade 1 was significantly different from that in normal controls (Figure 1(f)).

After detailed data analysis, we suggest that LRG1 is overexpressed in ccRCC patients, especially male Caucasian patients aged 40-80 with stage I or III disease. Therefore, LRG1 should be explored as a biomarker or target in ccRCC. To be more rigorous, a larger number of samples are needed to confirm these differences among subgroups of ccRCC patients classified by sex, age, race, stage, or grade.

### 3.3. LRG1 Is Related to the Prognosis of ccRCC

**Figure 2:**
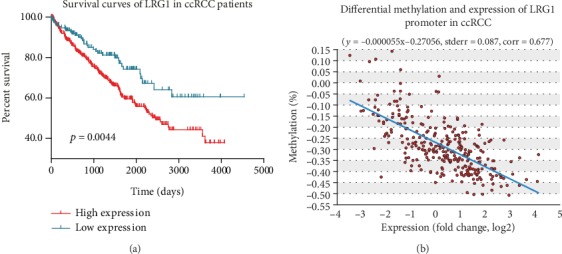
Survival curves and differential methylation and expression of LRG1 promoter in ccRCC patients. (a) Low LRG1 expression indicates a prolonged patient survival time. The FPKM cutoff value of high expression and low expression is 1.19. (b) The methylation level of LRG1 gene has a strong negative correlation (corr = 0.677) with the expression of LRG1.

The survival rate was determined by Kaplan-Meier survival curve analysis with Human Protein Atlas using RNA-seq data and clinical information from the TCGA. The Kaplan-Meier curve shows that LRG1 expression is closely related to ccRCC patient survival time and that low LRG1 expression indicates a prolonged patient survival time (*p* < 0.001) (Figure 2(a)). The median LRG1 expression was 2.88, and the median follow-up time was 3.28 years. The best cutoff value for mean fragments per kilobase of transcript per million mapped reads (FPKM) in the Kaplan-Meier survival curve analysis was 1.19, and 366 patients were classified as having high expression, while 158 patients were classified as having low expression. The FPKM value of low-expression patients was 0.54 ± 0.28, while that of high-expression patients was 7.61 ± 10.67.

### 3.4. Methylation of LRG1 Gene Is Downregulated in ccRCC Patients

**Figure 3:**
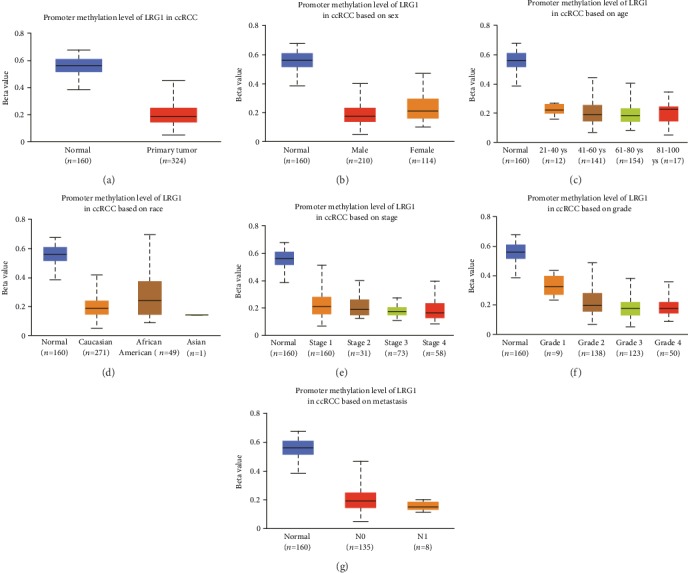
Promoter methylation level of LRG1 gene in ccRCC and subgroups. (a) Promoter methylation level of LRG1 gene is significantly downregulated compared with normal controls. (b) Methylation level of LRG1 gene in male and female patients is decreased compared with normal patients (*p* < 0.0001). (c) Methylation level of LRG1 gene in different ages has significant differences compared with normal controls (*p* < 0.0001). (d) Methylation level of LRG1 gene in different races has significant differences compared with normal controls (*p* < 0.001). (e) Methylation level of all of the stages are downregulated than normal controls (*p* < 0.001). (f) Methylation level of all of the grads are downregulated than normal controls (*p* < 0.0001). (g) Methylation level of metastatic ccRCC is lower than nonmetastatic, but both of them are significantly downregulated than normal controls (*p* < 0.001).

DNA methylation is the main form of epigenetic gene expression regulation in mammals. The methylation level of DNA is obviously decreased during the occurrence and development of many tumors. This decrease in the DNA methylation level makes gene expression active, which is one of the important ways that genes participate in promoting tumor development. The methylation level of LRG1 gene showed a strong negative correlation (corr = 0.677) with the expression of LRG1 (Figure 2(b)). The methylation of LRG1 gene was significantly downregulated in ccRCC tissue samples (*p* < 0.001) (Figure 3(a)). Methylation patterns in different subtype groups showed the same tendency (Figures 3(b)–3(g)). This indicates that a low methylation level upregulates the transcription of LRG1, thus accelerating the progression of ccRCC.

### 3.5. Verification

#### 3.5.1. LRG1 Expression Is Upregulated in ccRCC Tissue

**Figure 4:**
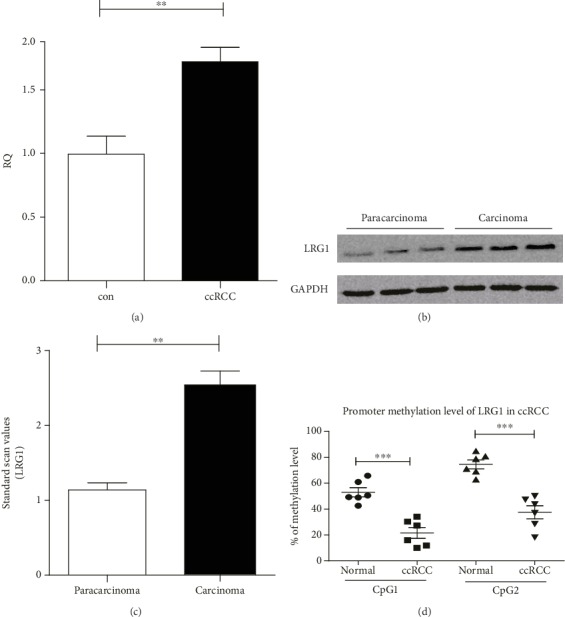
Expression and methylation level in carcinoma and paracarcinoma samples of ccRCC. (a) mRNA expression of LRG1 in carcinoma and paracarcinoma tissues (*p* < 0.01, *n* = 6) detected by qPCR. (b, c) Protein expression of LRG1 in carcinoma and paracarcinoma tissues (*p* < 0.01, *n* = 6) detected by western blot. (d) LRG1 methylation level of CpG1 and CpG2 is downregulated in ccRCC tissue (*p* < 0.0001).

To confirm the RNA-seq results for the TCGA data, LRG1 expression in 3 pairs of paracarcinoma and ccRCC carcinoma samples was determined by Western blotting and qPCR at the protein and mRNA levels, respectively. qPCR showed that LRG1 mRNA expression was significantly upregulated in the ccRCC carcinoma tissue samples (Figure 4(a)). Western blotting also showed significantly higher LRG1 expression in the carcinoma tissue samples than in the paracarcinoma tissue samples (Figures 4(b) and 4(c)).

#### 3.5.2. Methylation of LRG1gene Is Downregulated in ccRCC Tissue

We performed pyrosequencing with fresh paracarcinoma and carcinoma ccRCC samples collected at the PLA General Hospital to verify the differential methylation level observed for ccRCC patients in the TCGA database. Pyrosequencing showed that the methylation level was significantly downregulated (fold-change) in the carcinoma tissue samples compared with the paracarcinoma tissue samples (*p* < 0.01) (Figure 4(d)).

#### 3.5.3. LRG1 may Promote ccRCC Progression via the TGF-*β* Pathway

**Figure 5:**
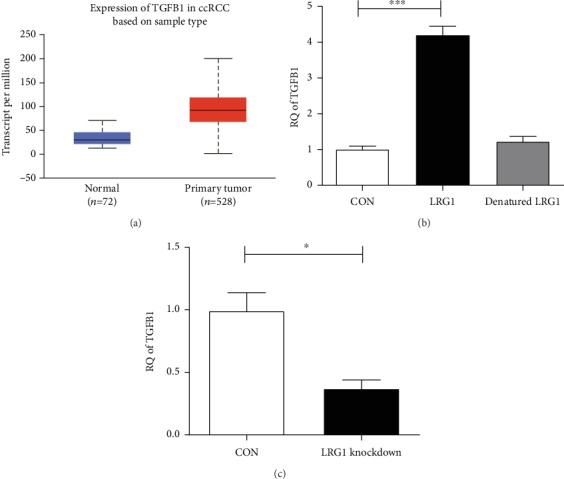
Expression of TGFB1 in ccRCC patients. (a) Expression of TGFB1 in ccRCC patients is upregulated (*p* < 0.001). (b) TGFB1 is upregulated in 789-O cells after stimulated with LRG1 (*p* < 0.001) but not with heat-denatured LRG1. (c) TGFB1 is downregulated in 789-O cells after LRG1 knockdown (*p* < 0.001).

A previous study showed that LRG1 promoted angiogenesis by modulating endothelial TGF-*β* signaling [5]. We also found that LRG1 promoted diabetic kidney disease progression by enhancing TGF-*β*–induced angiogenesis [6]. TCGA analysis showed that TGF-*β* expression was upregulated in ccRCC patients (Figure 5(a)). As a consequence, we speculate that LRG1 promotes ccRCC progression via the TGF-*β* pathway. In this study, we detected TGF-*β* mRNA expression in the 786-O cancer cell line after LRG1 stimulation and found that TGF-*β* expression was obviously increased (Figure 5(b)). Consistent with this finding, TGF-*β* expression was downregulated when LRG1 expression was knocked down by siRNA (Figure 5(c)).

## 4. Discussion

Our study showed the differential expression of LRG1 between ccRCC patients and normal controls. Both whole-cohort and subgroup analyses showed that LRG1 expression was upregulated significantly in ccRCC patients, especially in male Caucasian ccRCC patients. Moreover, survival time was negatively related to LRG1 expression. LRG1 has been identified to be differentially expressed in colorectal cancer, and a panel of the CEA, IGFBP2, MAPRE1, and LRG1 proteins is potentially a prediagnostic marker in colorectal cancer plasma samples [12]. All of these findings remind us that LRG1 is a potential novel pathogenic mediator in ccRCC.

Methylation is the main factor that regulates the transcription and expression of genes in cancer [13]. Methylated DNA protects the stability of the genome and reduces homologous recombination between repeated sequences, which can result in chromosome deletion and rearrangement. Reduced methylation of CpG islands in a promoter leads to overexpression of the genes controlled by the promoter. Methylation is closely associated with immunoproliferative diseases such as cancer [14–16], rheumatoid arthritis [17, 18], and proliferative diabetic retinopathy [5, 19] and even development and senescence [20, 21]. Once demethylation occurs at normally methylated sites, tumor progression is promoted. The methylation level of the LRG1 promoter was significantly decreased and negatively related to LRG1 expression, based on our TCGA data analysis and subsequent verification. Therefore, we hypothesized that overexpression of LRG1 caused by DNA demethylation promotes the progression of ccRCC.

What is the mechanism by which overexpressed LRG1 participates in tumor progression? LRG1 has a close relationship with TGF-*β*. Our previous work demonstrated that LRG1 significantly accelerated diabetic kidney injury and TGF-*β*/ALK1-induced angiogenesis in an experimental model of early diabetic kidney disease [6]. Consistently, LRG1 promotes angiogenesis by modulating endothelial TGF-*β* signaling in retinal vascular [5] and NSCLC [22]. LRG1 can also modulate the invasion and migration of glioma cell lines through the TGF-*β* signaling pathway [23]. Thus, we hypothesized that LRG1 promotes ccRCC progression via the TGF-*β* pathway based on this evidence. Evaluation of LRG1 cytokine stimulation and siRNA-mediated knockdown in 789-O cells confirmed our conjecture. In conclusion, we suggest that demethylation-induced overexpression of LRG1 accelerates ccRCC progression via the TGF-*β* pathway. To confirm this hypothesis more clearly, we are looking forward to more experiments and evidence.

## 5. Conclusion

In conclusion, LRG1 is overexpressed in ccRCC tissues, and low levels of LRG1 are closely related with a longer survival time in ccRCC patients rather than high LRG1 levels. Methylation level of LRG1 gene is significantly downregulated in ccRCC samples. Based on bioinformatics analysis and subsequent primary verification, we suggest that LRG1 may accelerate the progression of ccRCC via the TGF-*β* pathway. We are eager to determine the exact mechanism of LRG1 in more rigorous studies.

## Figures and Tables

**Table 1:** Pyrosequencing primer sequence.

Target	Forward	Reverse	Sequencing
hLRG1-CpG1	GTGGGGATTTTTTTAGGGTTGG	Bio-CTCCAAAAAAACATAATAACTCTACTCTT	GTTTAGGTAGGTATAAGGTTAT
hLRG1-CpG2	GATTTTTGGGGGGTATTTAAGAG	Bio-CCCTATCTCCAAAAATAATACCTTACA	ACCTTACAAACCTTAACC

**Table 2:** Clinical data of ccRCC patients.

	ccRCC	
Total	528	

Sex	Male	344
	Female	184
	Unclear	0

Age	Mean ± SD (years)	60.54 ± 12.14
	21-40	25
	41-60	239
	61-80	240
	81-100	23
	Unclear	1

Race	Caucasian	459
	African American	54
	Asian	8
	Unclear	8

Stage	I	263
	II	57
	III	123
	IV	82
	Unclear	4

Grade	1	14
	2	229
	3	206
	4	76
	Unclear	3

Survival	Alive	355
	Dead	173
	Unclear	0

Survival time		Days
	Median	1,195.5
	25%	545.25
	75%	1,928.25
	Min	2
	Max	4,537

## Data Availability

The data used to support the findings of this study are included within the article.
